# PKN1 Is a Novel Regulator of Hippocampal GluA1 Levels

**DOI:** 10.3389/fnsyn.2021.640495

**Published:** 2021-02-05

**Authors:** Motahareh Solina Safari, Dido Obexer, Gabriele Baier-Bitterlich, Stephanie zur Nedden

**Affiliations:** CCB-Biocenter, Institute of Neurobiochemistry, Medical University of Innsbruck, Innsbruck, Austria

**Keywords:** AMPA receptor, PKN1, NeuroD2, GluA1, hippocampus

## Abstract

Alterations in the processes that control α-Amino-3-hydroxy-5-methyl-4-isoxazolepropionic acid receptor (AMPAR) expression, assembly and trafficking are closely linked to psychiatric and neurodegenerative disorders. We have recently shown that the serine/threonine kinase Protein kinase N1 (PKN1) is a developmentally active regulator of cerebellar synaptic maturation by inhibiting AKT and the neurogenic transcription factor neurogenic differentiation factor-2 (NeuroD2). NeuroD2 is involved in glutamatergic synaptic maturation by regulating expression levels of various synaptic proteins. Here we aimed to study the effect of *Pkn1* knockout on AKT phosphorylation and NeuroD2 levels in the hippocampus and the subsequent expression levels of the NeuroD2 targets and AMPAR subunits: glutamate receptor 1 (GluA1) and GluA2/3. We show that PKN1 is expressed throughout the hippocampus. Interestingly, not only postnatal but also adult hippocampal phospho-AKT and NeuroD2 levels were significantly elevated upon *Pkn1* knockout. Postnatal and adult *Pkn1*^–/–^ hippocampi showed enhanced expression of the AMPAR subunit GluA1, particularly in area CA1. Surprisingly, GluA2/3 levels were not different between both genotypes. In addition to higher protein levels, we also found an enhanced GluA1 content in the membrane fraction of postnatal and adult *Pkn1*^–/–^ animals, while GluA2/3 levels remained unchanged. This points toward a very specific regulation of GluA1 expression and/or trafficking by the novel PKN1-AKT-NeuroD2 axis. Considering the important role of GluA1 in hippocampal development as well as the pathophysiology of several disorders, ranging from Alzheimer’s, to depression and schizophrenia, our results validate PKN1 for future studies into neurological disorders related to altered AMPAR subunit expression in the hippocampus.

## Introduction

We have recently identified the serine threonine kinase Protein kinase N1 (PKN1) as a developmentally active enzyme regulating axon growth, presynaptic maturation, and synapse formation in the Parallel fiber (PF)-forming cerebellar granule cells (Cgc). We discovered that PKN1-mediated AKT inhibition during critical stages of PF-maturation results in a reduction of the transcription factor neurogenic differentiation factor-2 (NeuroD2) and a subsequent increase in presynaptic specifications along PF. Consequently, *Pkn1* knockout leads to AKT hyperactivation as well as enhanced NeuroD2 protein levels, which results in a defective developmental synapse formation, a degeneration of cerebellar neurons and ataxia in adult animals (zur Nedden et al., 2018). *Neurod2* knockout animals exhibit morphological and physiological defects in various brain regions, including thalamocortical connections, hippocampal synaptogenesis, axonal guidance of callosal axons, development of amygdalar nuclei, cortical fasciculation, targeted axogenesis of compact fiber tracts as well as differences in intrinsic excitability during cortical development (Olson et al., 2001; Lin et al., 2005; Ince-Dunn et al., 2006; Wilke et al., 2012; Bormuth et al., 2013; Chen et al., 2016). Accordingly, disruption of NeuroD2 function has been implicated in several neurodevelopmental, neuropsychiatric and mood disorders, such as autism (Runge et al., 2020), depression (Bagot et al., 2016), schizophrenia (Spellmann et al., 2017), or epilepsy (Sega et al., 2019).

One striking role of NeuroD2 is the regulation of the subunit expression levels of ionotropic AMPARs. AMPARs are assemblies of four core subunits termed GluA1-4 (Collingridge et al., 2009), which mediate fast excitatory neurotransmission. Developmentally- and activity-regulated changes in AMPAR number and subunit composition are crucial for excitatory synapse formation, synaptic plasticity and neuronal circuit formation (Henley and Wilkinson, 2016). *Neurod2*^–/^*^–^* animals showed a marked reduction of GluA2/3 protein levels in layer IV of the cortex and cultured *Neurod2*^–/–^ neurons had a decreased surface expression of GluA1 and GluA2 subunits (Ince-Dunn et al., 2006). The effect of NeuroD2 was AMPAR-specific, since N-methyl-D-aspartate (NMDA) or kainate receptor expression was not affected. This implies that NeuroD2 controls the expression of AMPAR subunit proteins and/or proteins involved in the trafficking and surface retention of AMPARs. Indeed, NeuroD2 has been shown to regulate several genes involved in vesicle and receptor trafficking (Olson et al., 2001; Molnár and Molnár, 2006). Besides AMPARs, NeuroD2 has also been shown to regulate vesicular glutamate transporter 1 (VGlut1) (Bormuth et al., 2013), Synaptosomal-Associated Protein 25 kDa (SNAP-25) (Messmer et al., 2012), and postsynaptic density protein 95 (PSD-95) expression levels (Wilke et al., 2012).

The aim of this study was to analyze if PKN1 regulates hippocampal AKT and NeuroD2 and subsequently the protein levels and membrane-association of several synaptic proteins, with particular focus on GluA1 and GluA2/3.

## Materials and Methods

### Animals

The generation of *Pkn1* knockout mice (*Pkn1*^–/^*^–^* mice) has been described previously (Quétier et al., 2016). Animals were kindly provided by P. Parker and A. Cameron. Mice were backcrossed to C57BL/6N for more than 10 generations. C57BL/6N wildtype (WT) and C57BL/6N *Pkn1*^–/–^ animals were derived from the same heterozygous crosses and then bred separately, but kept under same housing and experimental conditions in the same room. C57BL/6N were derived from Jackson Laboratory. Animals younger than postnatal day (P)12 were killed by decapitation and animals older than P12 were killed by cervical dislocation. For studies in adult animals only 2–5 month old males were used.

### Preparation of Hippocampal Sections

After decapitation, brains were quickly removed, hemispheres were separated and a small block of tissue containing cortical and hippocampal regions was fixed in 4% PFA for 4–5 h.

#### Cryosections

After washing in PBS, hemispheres were incubated in 30% sucrose for a minimum of 24 h, embedded in optimal cutting temperature compound (Carl Roth) and stored at −80°C until analysis. 20 μm thick sagittal sections were cut with a cryostat (CM1950, Leica), transferred onto lysine-coated coverslips (Thermo Scientific) and allowed to dry for a minimum of 2 h at 37°C for further analysis or stored at −20°C. Sections were used for *in situ* hybridization.

#### Free-Floating Sections

After fixation hemispheres were washed in PBS and 50 μm thick sagittal sections were prepared with a Vibratome (VT1200S, Leica). Sections were used for antibody staining as described below.

### *In situ* Hybridization

*In situ* hybridization was performed employing the RNAScope Fluorescent Multiplex Assay kit (ACDBio). Cryosections from WT mice were dried (30 min, 60°C) and fixed for an additional 15 min in 4% PFA. Sections were processed as per manufacturers instructions, embedded in Mowiol (Sigma-Aldrich) and imaged with a widefield microscope (Axio, Axiocam 305, Zeiss and DMi8, Leica). The mean intensity of mRNA transcript was analyzed in Fiji (Schindelin et al., 2012) by tracing the dentate gyrus (DG) granule cell layer, CA3 and CA1 pyramidal cell layers. For comparability between experiments data was expressed as fold of the mean intensity of the DG for each experiment.

### Immunofluorescence Staining and Analysis of Mean GluA1 Intensity

Free-floating sections were subjected to antigen retrieval (10 mM sodium citrate, pH 6.0, 10 min at 100°C), washed in PBS, permeabilized (0.3% Triton-X-100, 45 min), blocked (10% goat serum, 2% BSA, 1 h), and primary antibodies (diluted in 0.1% Triton-X-100, 1% BSA, 5% goat serum in PBS) were added at room temperature overnight. After washing in PBS + 0.05% Tween for 30 min, sections were incubated with secondary antibodies (goat-anti rabbit Alexa-488 and goat anti-mouse Alexa-555) and Hoechst (8 μM) for 3 h at room temperature. After thorough washing in PBS + 0.05% Tween for 45 min, sections were placed on a microscope slide and embedded in Mowiol. To ensure comparable results all samples were processed on the same day using the same solutions. Images were taken with a widefield microscope (Axio, Axiocam 305, Zeiss), using the same exposure time, or a confocal microscope (SP8, Leica), using the same laser intensity for WT and *Pkn1*^–/–^ GluA1 stainings. Mean GluA1 intensity was analyzed in widefield images with Fiji by placing and measuring four 150 × 150 μm squares throughout CA1 stratum radiatum and oriens.

### Preparation of Subcellular Fractions and Western Blotting

For extraction of the detergent-soluble cytosolic and detergent-insoluble membrane fraction we followed the protocol of Takagi et al. (2010). Hippocampi of P12 or adult animals were carefully homogenized in TRIS-Buffer (20 mM Tris/HCl pH 7.5, 1 mM NaF, 10 μg/ml aprotinin, 10 μg/ml leupeptin, 50 μM PMSF, 2 mM sodium orthovanadate) and kept on ice for 30 min. After centrifugation (3000 × *g*, 10 min, 4°C) the supernatant was collected as cytosolic fraction. Pellets were washed once in TRIS-Buffer and resuspended in membrane extraction buffer (0.5% NP-40, 0.1% deoxycholate, 0.1% Brij 35, 10 mM DTT, 50 μM PMSF). Samples were incubated at 4°C for 60 min with gentle agitation. After centrifugation (10730 × *g*, 5 min, RT) supernatants were collected and resuspended in 4xLaemmli buffer (8% SDS, 40% glycerol, 20% β-mercaptoethanol, 0.01% bromophenol blue, and 250 mM Tris HCl, pH 6.8), boiled at 95°C for 5 min and stored at −20°C.

For whole cell extracts hippocampi were carefully dissected out in ice cold PBS and protein extraction was performed as described previously (zur Nedden et al., 2018). Protein contents were measured with a BCA assay kit (Thermo Fisher Scientific) and 35–70 μg protein was loaded onto self-made 10% or 12.5% continuous polyacrylamide gels. Western blotting was performed as described earlier (zur Nedden et al., 2018). Primary antibodies were added overnight in 5% BSA in TBS-T at 4°C and secondary antibodies (Li-Cor, anti-mouse 680 nm and anti-rabbit 800 nm) were added for 90 min at room temperature in 5% Milk in TBS-T. Membranes were imaged and analyzed with an Odyssey Clx infrared imager (Li-Cor). Stripping was not performed, however, for gels in Figures 3A,B a second blot in the same order was done to probe for PKN1, which would have otherwise masked the weak GluA1 signal. The correct separation of both fractions was assessed with appropriate loading controls.

### Antibodies

Clone numbers and RRID, where known, as well as catalog numbers (#) are provided in brackets. The following antibodies were from Cell signaling: AKT (40D4, #2920, RRID:AB_1147620), Na/K-ATPase (#3010, RRID:AB_2060983), GAPDH (D16H11, #5174, RRID:AB_10622025), phospho-AKT(T308) (D25E6, #13038, RRID:AB_2629447), PSD-95 (D27E11, #3450, RRID:AB_2292883 as well as 7E3, #36233, RRID:AB_2721262), Synapsin-1 (D12G5, #5297, RRID:AB_2616578), and VAMP2 (D601A, #13508, RRID:AB_2798240). VGlut1 (#48-2400, RRID:AB_2533843), goat anti-rabbit Alexa-488 (#A11070, RRID:AB_142134), and goat anti-mouse Alexa-555 (#A21425, RRID:AB_1500751) were purchased from Thermo Fisher Scientific. GluA1 (G-12, #sc-55509; RRID:AB_629532) and NeuroD2 (G-10, #sc-365896, RRID:AB_10843361) were from Santa Cruz. PKN1 (clone 49/PRK1, #610687, RRID:AB_398012) was from BD Transduction Laboratories. Actin (clone C4, #MAB1501, RRID:AB_2223041), VGlut2 (8G9.2, #ab79157, RRID:AB_1603114), tyrosine hydroxylase (#AB152, RRID:AB_390204), GluA2/3 (#AB1506, RRID:AB_90710), and GABA_A_β2/3 (#MAB341, RRID:AB_2109419) were from Merck Millipore. Znt3 (#197 011, RRID:AB_2189665) was purchased from Synaptic Systems. The secondary antibodies for the Odyssey infrared Imager, IR680 LT mouse (#92668020, RRID:AB_10706161) and IR800CW (#92632211, RRID:AB_621843) were purchased from Li-Cor. GABA_A_α4 was kindly provided by Gerald Obermair [a4N (1-14), (Hörtnagl et al., 2013)].

### Statistics

All data is presented as individual *n*-values with mean ± S.E.M., with *n*-values referring to different animals. For comparison of two independent groups a two-tailed unpaired *t*-test was used, for comparison of three or more groups a one-way ANOVA with Tukey’s multiple comparisons test was used and for comparison of two variables of two groups a two-way ANOVA was used. *P*-values smaller than 0.05 were considered as statistically significant. All analyses were performed in GraphPad prism 8.

## Results

### PKN1 Is Highly Expressed Throughout the Hippocampal Formation and Regulates AKT Phosphorylation and NeuroD2 Levels in Juvenile and Adult Animals

**FIGURE 1:**
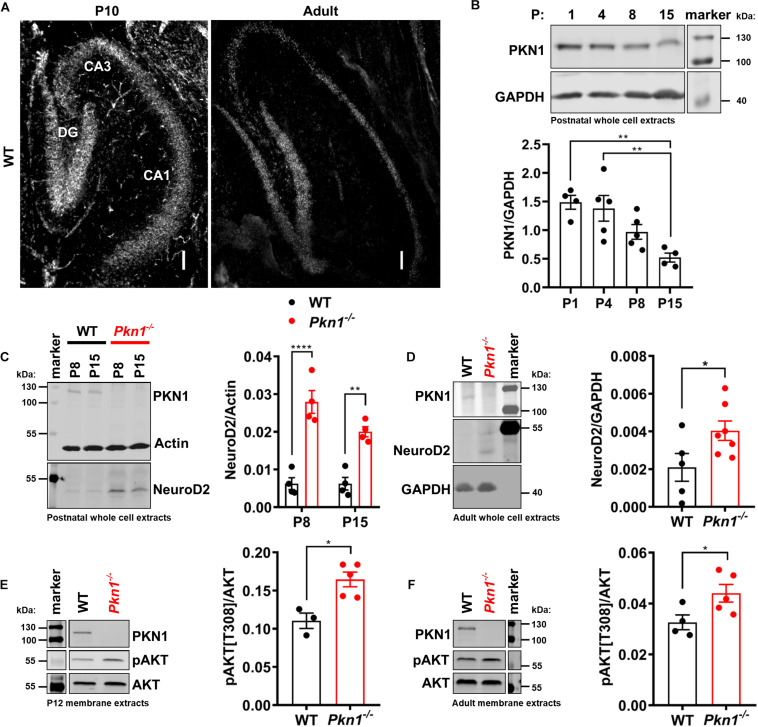
PKN1 regulates AKT phosphorylation and NeuroD2 levels in postnatal and adult hippocampi. **(A)** P10 and adult hippocampal sections from WT animals were tested for PKN1 expression by *in situ* hybridization. Pictures are representative of 2–3 separate WT animals. Scale bar refers to 100 μm. **(B)** PKN1 expression levels from P1 to P15 old WT hippocampi were assessed by western blotting (**P < 0.01, one way ANOVA with Tukey’s multiple comparisons test). **(C)** NeuroD2 expression in hippocampal whole cell protein extracts of P8 and P15 old WT and *Pkn1*^–/–^ animals was assessed by western blotting (two way ANOVA: Interaction: *P* = 0.0745, Age: *P* = 0.0747, Genotype: *P* < 0.0001, Sidak’s multiple comparisons test: P8: *P* < 0.0001, P15: *P* = 0.0025). **(D)** NeuroD2 expression in adult WT and *Pkn1*^–/–^ hippocampal whole cells extracts (*P = 0.0496, unpaired *t*-test). **(E)** Hippocampi from P12 old WT and *Pkn1*^–/–^ animals were separated into cytosolic and membrane fractions. Membrane extracts were probed for phosphorylated AKT[T308] (pAKT[T308]) and total AKT and the ratio was calculated (*P = 0.0104, unpaired *t*-test). **(F)** Membrane fractions of adult WT and *Pkn1*^–/–^ animals were probed for pAKT[T308] and AKT (*P = 0.045, unpaired *t*-test). The markers in the representative blots in **(E,F)** are shown in separate lanes as samples were not directly next to the markers in the blots. Data is presented as individual *n*-values with mean ± S.E.M.

Protein kinase N1 *in situ* hybridization revealed that PKN1 is abundantly expressed in the hippocampus in P10 old and adult animals. PKN1 mRNA was found in all hippocampal areas (Figure 1A). There was no difference in mean intensity levels between hippocampal layers in P10 old (mean intensity expressed as fold of DG mean intensity: 1 ± 00 for DG, 1.0 ± 0.08 for CA3, and 0.9 ± 0.01 for CA1, *n* = 3/genotype, *P* > 0.05, one way ANOVA, data not shown) or in adult animals (mean intensity expressed as fold of DG mean intensity: 1 for DG, 0.91 and 0.82 for CA3, 0.74 and 1.09 for CA1, *n* = 2/genotype, data not shown). Western blot analysis of WT whole cell hippocampal protein extracts revealed a significant reduction in PKN1 expression from P1 to P15 (Figure 1B) and a further decrease from P15 to adult animals (PKN1/Actin ratio was 0.026 ± 0.0007 for P15, *n* = 3, and 0.018 ± 0.0008 for adult WT animals, *n* = 4, ^∗∗^*P* = 0.0011, unpaired *t*-test, data not shown), suggesting an important role of PKN1 during postnatal development. The gross hippocampal morphology was not altered, and the layer thickness of all hippocampal layers was similar between both genotypes (Supplementary Figures 1A,B). The infrapyramidal mossy fiber projection was slightly enlarged upon *Pkn1* knockout (Supplementary Figures 1A,B). We did not detect aberrant sprouting of mossy fibers into the inner molecular layer of the DG in *Pkn1*^–/–^ animals (Supplementary Figure 1C). We next tested the effect of *Pkn1* knockout on hippocampal AKT phosphorylation and NeuroD2 levels. In agreement with our earlier findings in the cerebellum hippocampal NeuroD2 levels in postnatal *Pkn1*^–/–^ animals were strongly elevated at P8 and P15 (Figure 1C), suggesting that PKN1-mediated inhibition of NeuroD2 is important in various brain areas during development. Interestingly, we also observed an elevation of NeuroD2 levels in adult *Pkn1*^–/–^ hippocampi, even though this was less pronounced than during development (Figure 1D). AKT is primarily activated at the plasma membrane, and subsequently exerts its role at the plasma membrane or several subcellular compartments (Ebner et al., 2017; Lučić et al., 2018). To investigate if *Pkn1* knockout results in enhanced AKT phosphorylation, we prepared detergent-soluble (cytosolic) and detergent-insoluble fractions (membrane-associated proteins) of P12 old and adult WT and *Pkn1*^–/–^ hippocampi. The proper separation of fractions was assessed by appropriate loading controls. Importantly, the membrane-bound proteins Na^+^/K^+^-ATPase and PSD-95 were only found in the membrane extract (data not shown). We chose P12 as an intermediate age between P8 and P15. Interestingly we found that particularly membrane-associated AKT was hyper-phosphorylated upon *Pkn1* knockout in P12 old (Figure 1E) as well as in adult animals (Figure 1F). AKT phosphorylation in the cytosolic fraction was not different at both ages (Supplementary Figure 2). This contrasts with our findings in the cerebellum, where neither phospho-AKT nor NeuroD2 levels were different in adult animals, and suggests that this novel PKN1-AKT-NeuroD2 axis remains relevant for hippocampal function in adult animals.

### *Pkn1*^–/–^ Hippocampi Have Higher GluA1 Levels

**FIGURE 2:**
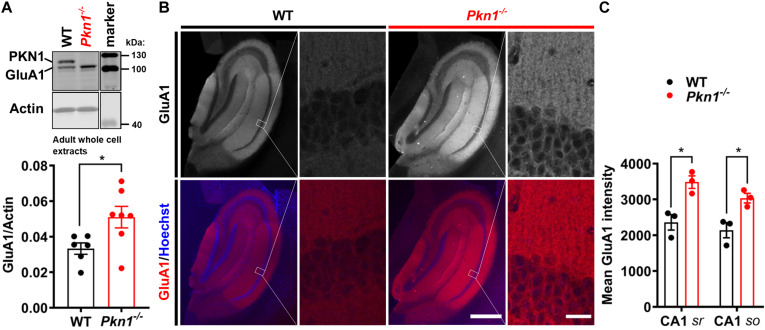
Adult *Pkn1*^–/–^ animals have enhanced GluA1 protein levels **(A)** Whole cell protein extracts from adult WT and *Pkn1*^–/–^ hippocampi were prepared, probed for GluA1 and the ratio to the loading control actin was calculated (*P = 0.0321, unpaired *t*-test). The marker in the representative blot is shown in a separate lane as samples were not directly next to the marker in the blot. **(B)** Hippocampal GluA1 levels were further assessed by immunofluorescence staining. Pictures are representative of 3 animals/genotype. Scale bar in overview images refers to 500 μm and scale bar in high resolution inserts refers to 20 μm. **(C)** The mean intensity of hippocampal GluA1 levels from **(B)** was quantified in Fiji. CA1 *sr* refers to CA1 stratum radiatum, CA1 *so* refers to CA1 stratum oriens. (CA1 *sr* *P = 0.0156, unpaired *t*-test, CA1 *so* *P = 0.0233, unpaired *t*-test). All data is presented as individual *n*-values with mean ± S.E.M.

It has been previously reported that *Neurod2* knockout animals show a reduction in GluA1, GluA2/3, PSD-95, and VGlut1 expression (Ince-Dunn et al., 2006; Wilke et al., 2012; Bormuth et al., 2013). Additionally, NeuroD2 controls the excitatory/inhibitory synaptic balance in pyramidal cortical neurons (Chen et al., 2016). We therefore probed adult hippocampal extracts from WT and *Pkn1*^–/–^ animals for a series of glutamatergic, gamma aminobutyric acid (GABA)-ergic and general synaptic markers (Figure 2 and Table 1). Adult *Pkn1*^–/–^ animals showed significantly elevated GluA1 levels (Figure 2A), however, GluA2/3 levels were not different between both genotypes (Table 1). Immunofluorescence staining revealed that GluA1 levels in *Pkn1*^–/–^ animals were particularly elevated in the CA1 area of the hippocampus (Figures 2B,C). Another protein significantly upregulated upon *Pkn1* knockout was VGlut 1 (Table 1), however, several other synaptic proteins (such as PSD-95, Znt-3, GABA_A_ receptor subtypes, see Table 1) were not, or only moderately affected by *Pkn1* knockout (such as SNAP-25).

**TABLE 1:** Expression levels of synaptic proteins in whole cell, cytosolic, and membrane fractions of P12 and adult animals.

	**WT (*n*-number)**	***Pkn1****^–^****^/^****^–^*** (*n*-number)**	***P*-Value**
**Adult whole cell extract**			
GluA2/3/Actin	0.145 ± 0.017 (6)	0.145 ± 0.014 (7)	0.836
VGlut1	0.003 ± 0.0005 (6)	0.005 ± 0.001 (7)	0.038 (*)
SNAP-25/Actin	0.572 ± 0.024 (6)	0.638 ± 0.020 (7)	0.0596
PSD-95/Actin	0.018 ± 0.002 (6)	0.019 ± 0.002 (7)	0.775
Znt-3/Actin	0.008 ± 0.002 (6)	0.011 ± 0.001 (7)	0.255
GABA_A_β2/3/Actin	0.000718 ± 0.002 (3)	0.00054 ± 0.0002 (4)	0.587
Tyrosine Hydroxylase/Actin	0.139 ± 0.026 (4)	0.128 ± 0.005 (4)	0.682
**P12 membrane/cytosolic fraction**			
VGlut1/Na/K-ATPase	0.000492 ± 0.0005 (3)	0.001075 ± 0.00009 (4)	0.012 (*)
VGlut2/Na/K-ATPase	0.456 ± 0.0798 (5)	0.641 ± 0.001 (4)	0.041 (*)
Membrane: GluA2/3/Actin^#^	0.037 ± 0.0066 (5)	0.038 ± 0.0052 (4)	0.815
Cytosol: GluA2/3/Actin	0.003 ± 0.0005 (5)	0.003 ± 0.0007 (4)	0.856
PSD-95/Na/K-ATPase	0.085 ± 0.007 (5)	0.103 ± 0.004 (4)	0.079
GABA_A_α4/Na/K-ATPase	0.101 ± 0.011 (5)	0.100 ± 0.022 (4)	0.968
GABA_A_β2/3/Na/K-ATPase	0.288 ± 0.043 (5)	0.391 ± 0.0345 (4)	0.117
Znt-3/Na/K-ATPase	0.002 ± 0.0004 (5)	0.003 ± 0.0002 (4)	0.124
**Adult membrane/cytosolic fraction**			
Membrane: GluA2/3/Actin^#^	0.2484 ± 0.025 (4)	0.2886 ± 0.037 (5)	0.425
Cytosol: GluA2/3/Actin	0.0218 ± 0.003 (4)	0.019 ± 0.003 (5)	0.537
PSD-95/Na/K-ATPase	28.6 ± 1.65 (4)	25.3 ± 1.80 (5)	0.226
GABA_A_α4/Na/K-ATPase	0.37 ± 0.03 (4)	0.34 ± 0.04 (5)	0.517
Znt-3/Na/K-ATPase	0.44 ± 0.03 (4)	0.49 ± 0.02 (5)	0.114
VAMP2/Na/K-ATPase	6.07 ± 0.83 (4)	7.30 ± 0.91 (5)	0.362
Synapsin 1/Na/K-ATPase	3.45 ± 0.22 (4)	4.08 ± 0.27 (5)	0.122

Whole cell extracts and cytosolic extracts are expressed in relation to the loading controls actin. Membrane extracts are expressed in relation to the loading control Na^+^/K^+^-ATPase. ^#^Since GluA2/3 and Na^+^/K^+^-ATPase had the same size (∼100 kDa) and host species we used Actin as a loading control for both fractions. (*) *P* < 0.05, unpaired *t*-test.

### *Pkn1*^–/–^ Animals Show Higher Membrane-Associated GluA1 Levels

**FIGURE 3:**
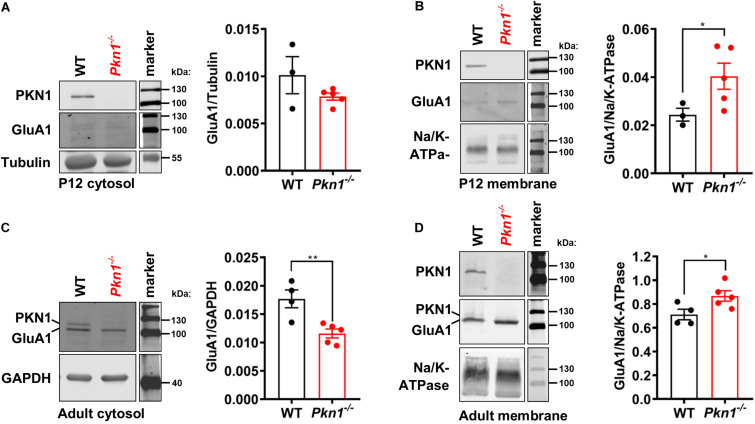
Postnatal and adult *Pkn1*^–/–^ animals show enhanced membrane-association of GluA1. **(A)** The detergent-soluble cytosolic fraction of extracts prepared from P12 old WT and *Pkn1*^–/–^ hippocampi was probed for the loading control Tubulin and GluA1 (*P* = 0.393, unpaired *t*-test). **(B)** The detergent-insoluble membrane fraction of extracts prepared from P12 old WT and *Pkn1*^–/–^ hippocampi was probed for the loading control Na^+^/K^+^-ATPase and GluA1 (*P = 0.0416, unpaired *t*-test). **(C)** The detergent-soluble cytosolic fraction of extracts prepared from adult WT and *Pkn1*^–/–^ hippocampi was probed for the loading control GAPDH and GluA1 (**P = 0.0075, unpaired *t*-test). **(D)** The detergent-insoluble membrane fraction of extracts prepared from adult WT and *Pkn1*^–/–^ hippocampi was probed for the loading control Na^+^/K^+^-ATPase and GluA1 (*P = 0.0430, unpaired *t*-test). All data is presented as individual *n*-values with mean ± S.E.M. Images of PKN1 in **(A,B)** were derived from a second blot, loaded in the same order. All markers in the representative blots are shown in separate lanes as samples were not next to the markers in the blots.

In addition to decreased protein expression levels, *Neurod2* knockout neurons showed a reduction in GluA1 surface expression (Ince-Dunn et al., 2006). To test if hippocampal extracts from young postnatal and adult animals show differences in cytosolic and membrane-associated GluA1 levels, we analyzed the detergent-soluble cytosolic [contains soluble cytosolic enzymes, as well as, due to the low g force and centrifugation duration transport/recycling vesicles (Jeppesen et al., 2014)] and the detergent-insoluble membrane protein fraction (contains plasma membrane-associated proteins and vesicles) of P12 old and adult WT and *Pkn1*^–/–^ hippocampi by immunoblotting. While we did not find a significant difference in the GluA1 content in the cytosolic fraction of P12 old animals (Figure 3A), we observed a significant increase in GluA1 levels in the membrane fraction of *Pkn1*^–/–^ hippocampi (Figure 3B). In accordance with our results in adult animals we found that VGlut1 and VGlut2 were significantly elevated upon *Pkn1* knockout in P12 old animals (Table 1). In adult *Pkn1*^–/–^ animals GluA1 levels in the cytosolic fraction were significantly reduced (Figure 3C) while the membrane-associated content of GluA1 was significantly increased (Figure 3D). This suggests that besides differences in protein levels, GluA1 trafficking might be affected by *Pkn1* knockout in adult animals. GluA2/3 levels in the cytosolic or membrane fraction of P12 old and adult animals were not different between both genotypes (Table 1). Likewise, the content of several other synaptic proteins (such as PSD-95, synapsin 1, Znt-3, GABA_A_ Receptor subtypes) was not significantly affected by *Pkn1* knockout (Table 1).

## Discussion

Here we establish PKN1 as a novel regulator of hippocampal GluA1 levels and GluA1 membrane/cytosol trafficking. Mechanistically we provide evidence that phosphorylated AKT and the transcription factor NeuroD2, which has been shown to regulate AMPAR subunit expression and membrane insertion (Olson et al., 2001; Ince-Dunn et al., 2006; Molnár and Molnár, 2006; Wilke et al., 2012), are strongly elevated upon *Pkn1* knockout in postnatal and adult animals.

We have recently shown that PKN1 regulates NeuroD2 levels in an AKT-dependent manner, thereby controlling the precise balance between axonal growth and presynaptic differentiation in early postnatal cerebellar development (zur Nedden et al., 2018). Accordingly, *Pkn1*^–/–^ animals showed AKT hyperphosphorylation, higher NeuroD2 protein levels and subsequently a decrease in presynaptic specifications accompanied by a defective PF-Purkinje cell synapse formation. The fact that the hippocampus shows a similar dysregulation of AKT and NeuroD2 upon *Pkn1* knockout suggests that the tight control of postnatal AKT/NeuroD2 levels by PKN1 constitutes a general and important regulatory mechanism in the development of several brain areas. However, we also observed significantly increased AKT phosphorylation and NeuroD2 levels in adult *Pkn1*^–/–^ hippocampi. This contrasts our results obtained in the cerebellum, where neither phospho-AKT nor NeuroD2 were elevated in adult animals, and shows that PKN1-mediated AKT and NeuroD2 inhibition remains important in the hippocampus beyond postnatal development.

*Pkn1*^–/–^ animals showed a specific elevation of hippocampal GluA1 levels, while GluA2/3 levels as well as other NeuroD2 downstream targets such as PSD-95 were not altered. These findings are surprising, since it was reported that NeuroD2 controls both, thalamocortical GluA1/2/3 (Ince-Dunn et al., 2006) as well as hippocampal PSD-95 expression and mossy fiber-CA3 synaptic maturation (Wilke et al., 2012). We have not analyzed mossy fiber-CA3 synapse number or CA3-specific PSD-95 expression, which might reveal more subtle differences between WT and *Pkn1*^–/–^ animals. Nevertheless, a possible explanation for these apparent differences might be that PKN1 controls NeuroD2 activity in a very targeted manner. This is further supported by the fact that only very few synaptic downstream targets of NeuroD2 were elevated upon *Pkn1* knockout, such as VGlut1. Besides PKN1, the activity/expression of NeuroD2 is controlled by other basic helix-loop-helix factors (neurogenin 1) (Lin et al., 2004), various signaling cascades and posttranslational modifications, such as phosphorylation (Dennis et al., 2019). Furthermore, calcium acts as an activity-dependent regulator of NeuroD2 (Ince-Dunn et al., 2006). Therefore, an altered synaptic protein content could have a secondary influence on NeuroD2-mediated transcription upon *Pkn1* knockout. Moreover, there are brain area-specific differences in the effect of NeuroD2 overexpression versus deficiency on synaptic maturation. In the cerebellum, our (zur Nedden et al., 2018) and an earlier report (Yang et al., 2009) showed that in Cgc, NeuroD2 levels are high during axonal/dendritic growth, where it prevents premature synaptogenesis but need to be degraded in order to drive presynaptic maturation of PFs. On the contrary, *Neurod2*-deficient Cgc functionally integrate into the cerebellar circuit (Pieper et al., 2019), suggesting that NeuroD2 is redundant and/or not essential for correct PF-synaptic maturation. In the hippocampus, however, *Neurod2*-deficiency results in reduced expression of PSD-95 and a defective CA3-mossy fiber synapse formation, establishing NeuroD2 as a non-redundant and important regulator of synaptic maturation in that brain area (Wilke et al., 2012). Therefore, *Neurod2*-deficiency versus overactivation might not be directly comparable and result in diverse outcomes in different brain areas.

Besides higher GluA1 levels we also found an elevated GluA1 content in the membrane fraction of postnatal and adult *Pkn1*^–/–^ animals. While we cannot deduce if this is related to enhanced surface expression of GluA1, it points toward enhanced transportation of GluA1 to the plasma membrane. Additionally, adult animals showed significantly lower GluA1 levels in the cytosolic fraction (which contains transport/recycling vesicles), suggesting a potential difference in GluA1 trafficking. NeuroD2 regulates different populations of target genes in the embryonal and postnatal cortex (Guzelsoy et al., 2019). Therefore, the difference between P12 and adult animals might lie in differently regulated downstream genes involved in receptor trafficking and surface retention (Olson et al., 2001; Molnár and Molnár, 2006). Alternatively, PKN1 itself might be involved in the regulation of GluA1 trafficking and/or degradation. PKN1 belongs to the PKC superfamily, and PKCs as well as PKA are well known for their roles in phosphorylation of GluA1, thereby regulating channel conductance, internalization, and receptor trafficking (Buonarati et al., 2019).

The functional implications of enhanced GluA1 protein levels/membrane-association in *Pkn1*^–/–^ animals remain to be elucidated. AMPAR subunit composition determines the conductance, trafficking and calcium permeability of these receptors. Calcium permeability is mainly conferred by the presence or absence of the calcium impermeable GluA2 subunit. Hippocampal AMPAR primarily exist as either GluA1/2 (>80%) or GluA2/3 heteromers (Lu et al., 2009). Calcium permeable (CP) GluA1 homomers are particularly abundant in immature CA1 synapses (Stubblefield and Benke, 2010; Henley and Wilkinson, 2016; Benke and Traynelis, 2019). Although less frequent, CP-AMPAR are also found in adult neurons where they contribute to the induction of long term potentiation, to homeostatic synaptic scaling and potentially long term depression (Henley and Wilkinson, 2016). Defects in the regulation of AMPAR subunit expression, assembly, trafficking, and membrane insertion are closely linked to psychiatric conditions as well as cognitive decline and neurodegenerative diseases, including Alzheimer’s disease (Walsh and Selkoe, 2007; Whitcomb et al., 2015) or mood disorders (Naylor et al., 1996; Martinez-Turrillas et al., 2002; Du et al., 2008). Addition of only 5% homomeric GluA1 to the pool of AMPAR in hippocampal neurons can already account for a conductance change (Guire et al., 2008). Therefore, it would be interesting to study if and how extrasynaptic and synaptic AMPAR composition is affected by the enhanced GluA1 protein levels upon *Pkn1* knockout.

Recently it was shown that PKN1 promotes synapse maturation by inhibiting type I metabotropic glutamate receptor-dependent long term depression through regulation of excitatory amino acid transporter 3 (EAAT3) in area CA1 (Yasuda et al., 2020). Knockdown of PKN1 to 1/10th of WT levels results in immature synaptic transmission, more silent synapses and fewer spines with shorter postsynaptic densities in juvenile CA1 neurons. While there might be a difference between our complete *Pkn1* knockout animals and the reduction of PKN1 to 1/10th of WT animals, this report would imply that (1) enhanced protein levels of GluA1 and VGlut1 are not due to an increase in synapse number, which is further supported by unchanged PSD-95 levels, but rather reflect a selective increase in protein concentrations of these specific synaptic proteins; and (2) enhanced GluA1 expression might not be translated into a higher synaptic content of GluA1 homomers. However, authors did not analyze AMPAR subunit composition or adult *Pkn1* knockdown animals, and considering EAAT3 expression drops during development (Bjørn-Yoshimoto and Underhill, 2016), electrophysiological properties in adult *Pkn1*^–/–^ or *Pkn1* knockdown animals might reveal differences in AMPAR function and/or composition.

Taken together our data validate PKN1 as a novel regulator of hippocampal GluA1 levels and GluA1 membrane-association, thereby providing a new tool to understand the functional consequences of this specific subunit as well as raising the potential for the modulation of GluA1 as a possible strategy for therapeutic intervention. In that context, it is worth noting that non-specific PKN1 inhibitors have already been validated for safe use in humans (Shibuya et al., 2005; Cohen et al., 2017) and the development of specific PKN1 inhibitors is a focus of cancer research (Ostrovskyi et al., 2016).

## Data Availability Statement

The raw data supporting the conclusions of this article will be made available by the authors, without undue reservation.

## Ethics Statement

Ethical review and approval was not required for the animal study because they are classified as *in vitro*. However, we are monitored and certified (FELASA) by Austrian authorities with regard to animal handling, cervical dislocation, and decapitation. Every effort was taken to minimize the number of animals used. Written informed consent was obtained from the owners for the participation of their animals in this study.

## Author Contributions

SzN, MS, and GB-B developed the study concept and design. MS, DO, and SzN performed immunoblotting, immunohistochemistry, and analysis of all data. MS and SzN prepared the figures. SzN and GB-B supervised the project and wrote the manuscript with critical input from all authors. All authors approved the manuscript.

## Conflict of Interest

The authors declare that the research was conducted in the absence of any commercial or financial relationships that could be construed as a potential conflict of interest.

## Abbreviations

AMPAR: α -amino-3-hydroxy-5-methyl-4-isoxazolepropionic acid receptor
A β: amyloid- β
BSA: bovine serum albumin
Cgc: cerebellar granule cells
CA1-3: Cornu Ammonis area 1-3
DG: dentate gyrus
EAAT3: excitatory amino acid transporter 3
GABA: gamma aminobutyric acid subtype A (GABA_A_) receptors
GAPDH: glyceraldehyde 3-phosphate dehydrogenase
GluA1: glutamate receptor 1
GluA2/3: glutamate receptor 2/3
HCl: hydrochloric acid
NeuroD2: neurogenic differentiation factor-2
NMDA: N-methyl -D-aspartate
PFA: paraformaldehyde
PF: Parallel fiber
P: postnatal day
PBS: phosphate buffered saline
pAKT: phosphorylated AKT
PMSF: phenylmethylsulfonyl fluoride
PKN1: Protein kinase N1
PSD-95: postsynaptic density protein 95
SDS: Sodium dodecyl sulfate
NaF: sodium fluoride
SNAP-25: Synaptosomal-Associated Protein 25 kDa
TBS-T: Tris buffered saline with Tween 20
VAMP2: vesicle associated membrane protein 2
VGlut 1 and 2: vesicular glutamate transporter 1 and 2.
