# Supplementary material for: PKN1 Is a Novel Regulator of Hippocampal GluA1 Levels
Source: Front Synaptic Neurosci. 2021 Feb 5;13:640495. doi: 10.3389/fnsyn.2021.640495 (PMC7892898; doi:10.3389/fnsyn.2021.640495)
Supplement: Supplementary file 1 [file Data_Sheet_1.pdf]

Supplementary methods:

Analysis of hippocampal mossy fiber morphology

For P10 calbindin staining fixated brains were embedded in paraffin. 5 µm thick sagittal sections were cut and allowed to dry overnight. After deparaffinization, antigen retrieval at pH 9 was performed (10 mM Tris base, 1 mM EDTA solution, pH 9.0). For calbindin staining of adult hippocampi free-floating 50 µm thick sections were prepared with a Vibratome (VT1200S, Leica) in PBS.

Hippocampal mossy fibers (MF) were stained with C1ql3 (P10), calbindin (P10 and adult) or Znt-3 (adult). 2-3 sections per animal from equivalent rostral-caudal locations were selected using anatomical landmarks and the size and shape of the hippocampus. The area of the infra- and intrapyramidal (IP) MF (measured from the exit of the hilus to the furthest visible staining in the stratum oriens) and the area of the suprapyramidal (SP) MF (measured from the exit of the hilus to the apex of the curvature of the CA3 pyramidal cell layer in the stratum lucidum) was measured. For all analyses Fiji was used (Schindelin et al., 2012). Calbindin was purchased from cell signaling (D1I4Q, #13176, RRID: AB_2687400) and C1ql3 (#230660) was from Abcam.

Analysis of hippocampal layer thickness

1-3 Hoechst-stained sections per animal from equivalent rostral-caudal locations were selected using anatomical landmarks and the size and shape of the hippocampus. DG granule cell layer thickness was measured at 5 locations/section of the outer and 5 locations/section of the inner blade and the mean was calculated. CA3 and CA1 pyramidal cell layer thicknesses were measured at 5 locations/section and the mean was calculated. The mean of all analyzed sections/animal is shown. For all analyses Fiji was used.

References

Schindelin, J., Arganda-Carreras, I., Frise, E., Kaynig, V., Longair, M., Pietzsch, T., et al. (2012). Fiji: an open-source platform for biological-image analysis. *Nature Methods* 9(7)**,** 676-682. doi: 10.1038/nmeth.2019.

Supplementary Figure legends:

Supplementary Figure 1: *Pkn1$^{-/-}$* animals have an enlarged infrapyramidal mossy fiber bundle. (**A**) MF in hippocampal sections of P10 old WT and *Pkn1$^{-/-}$* animals were stained with Calbindin (green) and Hoechst (nuclei, blue). Pictures are representative of 3 animals per genotype. Scale bar refers to 100 µm. Calculation of the IP MF and SP MF area of hippocampal sections from P10 old WT and *Pkn1$^{-/-}$* animals stained with C1ql3 or Calbindin (**P=0.0097, unpaired t-test). The thickness of all hippocampal regions was not different between both genotypes (P>0.05, unpaired t-test). (**B**) Calbindin staining revealed an enlarged IP MF area in adult *Pkn1$^{-/-}$* animals. Scale bar refers to 200 µm. Calculation of the IP and SP MF area of hippocampal sections from adult WT and *Pkn1$^{-/-}$* animals stained with Znt-3 or Calbindin (* P=0.0309, unpaired t-test). The thickness of all hippocampal regions was not different between both genotypes (P>0.05, unpaired t-test). (**C**) Znt-3 staining revealed no MF sprouting in adult animals. Images are representative of at least 3 animals/genotype. Scale bar refers to 200 µm. All data is presented as individual *n*-values with mean ± S.E.M.

Supplementary Figure 2: Phosphorylated AKT levels in the cytosolic fraction are not different between WT and *Pkn1$^{-/-}$* animals. (**A**) Hippocampi from P12 old WT and *Pkn1$^{-/-}$* animals were separated into cytosolic and membrane fractions. Extracts were probed for pAKT[T308] and total AKT and the ratio was calculated. There was no difference in pAKT[T308]/AKT in the cytosolic fraction (P=0.8413, unpaired t-test). (**B**) Hippocampi from adult WT and *Pkn1$^{-/-}$* animals were separated into cytosolic and membrane fractions. Extracts were probed for pAKT[T308] and total AKT and the ratio was calculated. There was no difference in pAKT[T308]/AKT in the cytosolic fraction (P=0.247, unpaired t-test). For membrane fractions refer to Figure 1 E, F. All markers in the representative blots are shown in separate lanes as samples were not next to the markers in the blots. All data is presented as individual *n*-values with mean ± S.E.M.

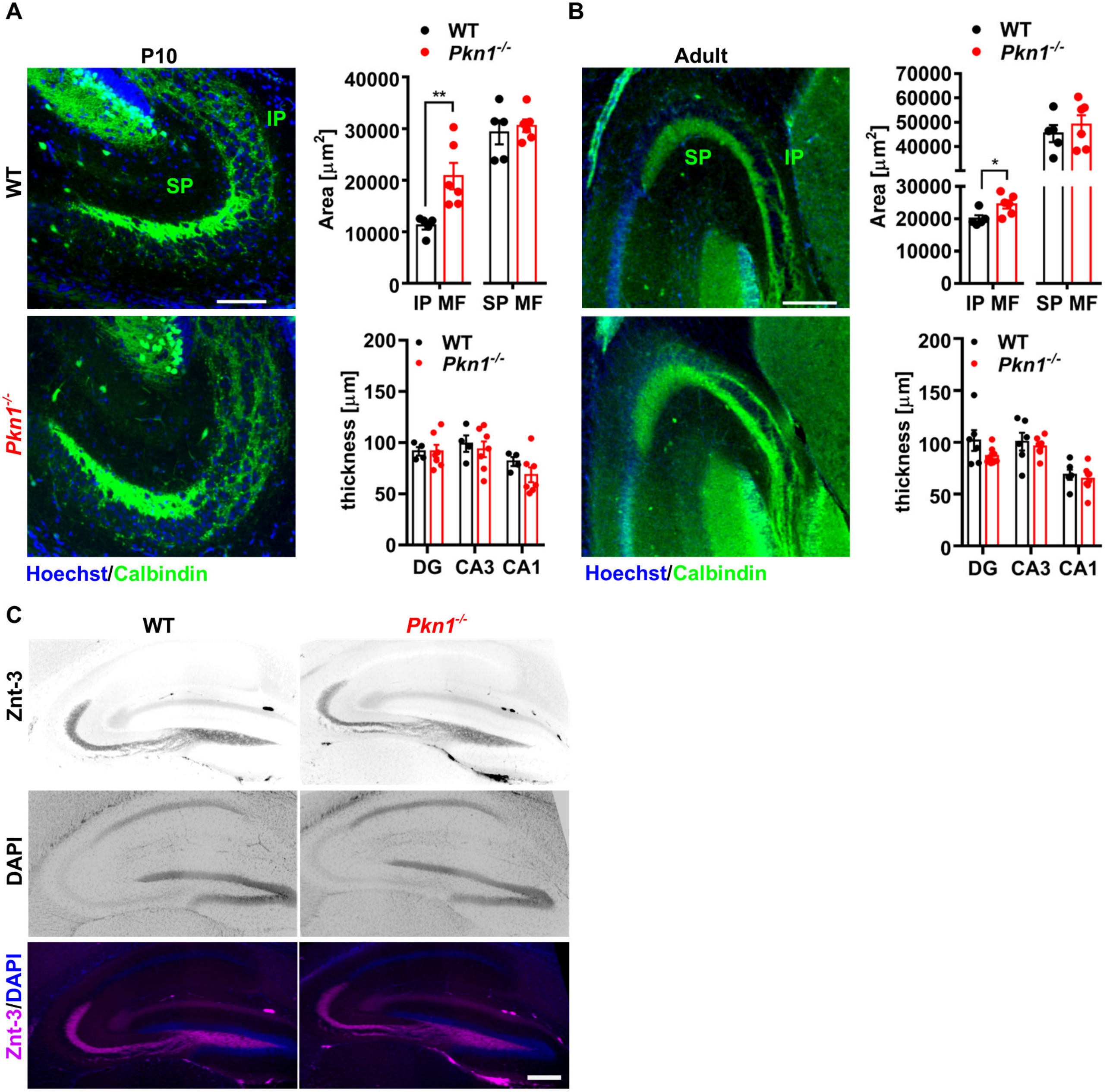

Supplementary Figure 1

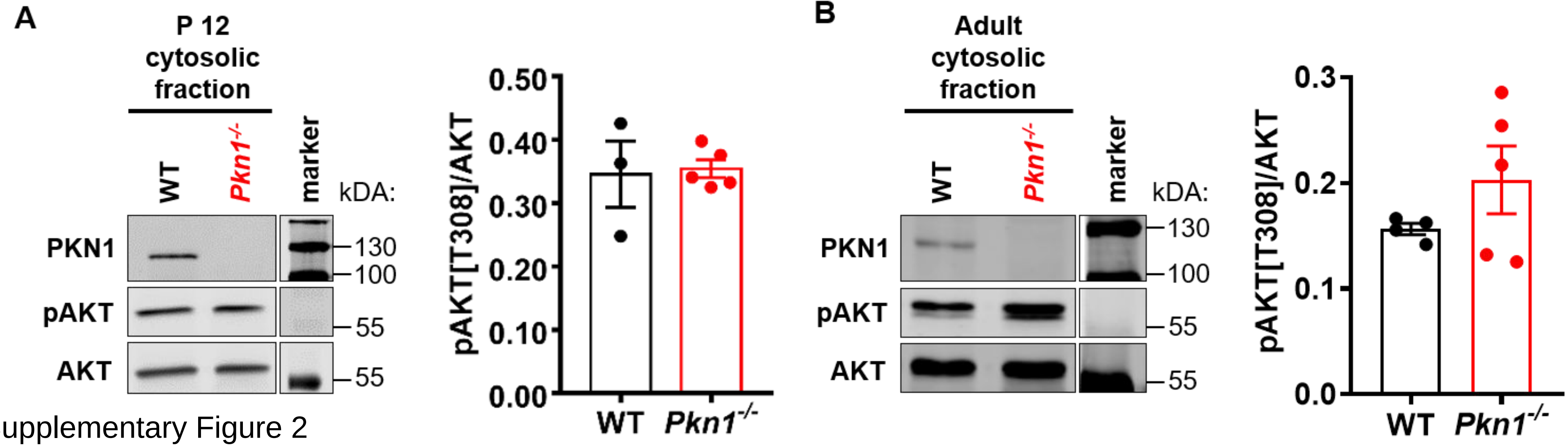

Supplementary Figure 2
